# Benefit of early discharge among patients with low-risk pulmonary embolism

**DOI:** 10.1371/journal.pone.0185022

**Published:** 2017-10-10

**Authors:** Li Wang, Onur Baser, Phil Wells, W. Frank Peacock, Craig I. Coleman, Gregory J. Fermann, Jeff Schein, Concetta Crivera

**Affiliations:** 1 STATinMED Research, Plano, Texas, United States of America; 2 Center for Innovation & Outcomes Research, Department of Surgery, Columbia University and STATinMED Research, New York, New York, United States of America; 3 MEF University, Department of Economics, Istanbul, Turkey; 4 Department of Medicine, University of Ottawa and the Ottawa Hospital Research Institute, Ottawa, Ontario, Canada; 5 Emergency Medicine, Baylor College of Medicine, Houston, Texas, United States of America; 6 School of Pharmacy, University of Connecticut, Hartford, Connecticut, United States of America; 7 Department of Emergency Medicine, University of Cincinnati, Cincinnati, Ohio, United States of America; 8 Janssen Scientific Affairs, LLC, Titusville, New Jersey, United States of America; National and Kapodistrian University of Athens, GREECE

## Abstract

Clinical guidelines recommend early discharge of patients with low-risk pulmonary embolism (LRPE). This study measured the overall impact of early discharge of LRPE patients on clinical outcomes and costs in the Veterans Health Administration population. Adult patients with ≥1 inpatient diagnosis for pulmonary embolism (PE) (index date) between 10/2011-06/2015, continuous enrollment for ≥12 months pre- and 3 months post-index date were included. PE risk stratification was performed using the simplified Pulmonary Embolism Stratification Index. Propensity score matching (PSM) was used to compare 90-day adverse PE events (APEs) [recurrent venous thromboembolism, major bleed and death], hospital-acquired complications (HACs), healthcare utilization, and costs among short (≤2 days) versus long length of stay (LOS). Net clinical benefit was defined as 1 minus the combined rate of APE and HAC. Among 6,746 PE patients, 95.4% were men, 22.0% were African American, and 1,918 had LRPE. Among LRPE patients, only 688 had a short LOS. After 1:1 PSM, there were no differences in APE, but short LOS had fewer HAC (1.5% vs 13.3%, 95% CI: 3.77–19.94) and bacterial pneumonias (5.9% vs 11.7%, 95% CI: 1.24–3.23), resulting in better net clinical benefit (86.9% vs 78.3%, 95% CI: 0.84–0.96). Among long LOS patients, HACs (52) exceeded APEs (14 recurrent DVT, 5 bleeds). Short LOS incurred lower inpatient ($2,164 vs $5,100, 95% CI: $646.8-$5225.0) and total costs ($9,056 vs $12,544, 95% CI: $636.6-$6337.7). LRPE patients with short LOS had better net clinical outcomes at lower costs than matched LRPE patients with long LOS.

## Introduction

### Background

Pulmonary embolism (PE) is responsible for at least 100,000–200,000 deaths in the United States each year, and is the third major cause of cardiovascular death after myocardial infarction and cerebrovascular accidents [1]. The annual incidence rate of PE is 1.0 per 1,000 persons, rising exponentially with age [2]. Based on autopsy data results, incidence of this disease is highest among individuals aged between 70–80 years. If untreated, acute PE is associated with a death rate as high as 30%, whereas the death rate of diagnosed and treated PE is 8% [3].

### Importance

PE is generally diagnosed in patients by performing a Computed Tomography Angiography (CTA), and the majority of US patients are admitted for risk stratification and initiation of anticoagulation therapy [4]. Several risk-stratification algorithms have been developed, including the Geneva score, the Pulmonary Embolism Severity Index (PESI) score, the simplified PESI (sPESI) score, the Spanish score, the Davies criteria, the Home management exclusion criteria, and the Hestia criteria [5,6].

PE is associated with a substantial burden of health care utilization and associated costs. The annual cost per patient for an initial episode of PE ranges from $13,000 –$31,300, and with recurrent episodes, the annual cost per patient is $11,014 –$14,722 [7]. Since patients with low-risk PE (LRPE) can be identified using the validated risk stratification tools, an opportunity exists to select a population of patients that can be safely treated without a traditional hospital admission [7]. It has been estimated that up to 50% of PE patients can be treated safely in an outpatient setting [8]. American and European professional organizations have guidelines that support such an approach. The updated 2016 ACCP guidelines suggest that LRPE patients with adequate home circumstances can be treated at home or with an abbreviated hospital stay [8]. The European Society of Cardiology advocates for the risk stratification of PE patients and the consideration of an outpatient management option for LRPE patients [9,10]. While treatment of LRPE patients in outpatient settings is widely practiced in European countries, physicians in the US have not widely adopted an outpatient or observation management strategy. Some factors identified as barriers to outpatient management of LRPE patients include physician resistance, medication security, difficulty in risk stratification, and lack of a uniform approach to risk stratification [11].

### Goals of this investigation

Given the numerous studies supporting the benefit of early discharge among LRPE patients, the purpose of our study was to examine whether short length of stay (LOS) is associated with improved clinical and economic outcomes in a real-world setting.

## Methods

### Study design and setting

This was a longitudinal, retrospective cohort study assessing the Veterans Health Administration (VHA) population from October 1, 2010 through September 30, 2015. The VHA is the largest integrated health care system in the United States, providing care at 1,245 health care facilities, including 170 VA medical centers and 1,065 outpatient clinics, serving more than 9 million enrolled veterans across the country [12]. Since this study does not involve the collection, use or transmittal of individual identifiable data, Institutional Review Board approval to conduct this study was not required.

Electronic health data collected within the VHA national Medical SAS^®^ Dataset and Decision Support System were evaluated, using the medical, pharmacy, laboratory, and VHA health plan enrollment information [13,14]. These data include hospital and outpatient diagnoses (International Classification of Diseases, 9th Revision, Clinical Modification [ICD-9-CM]) and procedure codes (ICD-9 procedure and Current Procedural Terminology codes), [15] laboratory results, and dispensed medication records. Death date was determined using the VA Vital Status file, which ascertains mortality using the Social Security Death Master File, Medicare Vital Status Files, and VA Beneficiary Identification and Records Locator Subsystem. The VHA Vital Status File is updated quarterly, and the three most recent quarterly updates are maintained [16].

#### Selection of participants

Patients were included in the study if they were ≥18 years of age, had ≥1 inpatient diagnosis for PE (ICD-9-CM codes: 415.1, 415.11, 415.19), had a prescription claim for an anticoagulant (unfractionated heparin, low-molecular-weight heparin [LMWH], warfarin, or novel oral anticoagulants [NOACs]) during the hospital stay and were continuously enrolled in a health plan with medical and pharmacy benefits for at least 12 months prior to the index hospitalization discharge, including the hospital stay (baseline period) until 3 months post-index date or death (follow-up period), whichever came first. The first PE diagnosis date was designated as the initial diagnosis date, and the discharge date was designated as the index date. Patients who were administered subcutaneous heparin during the hospital stay were not included, since many patients are given subcutaneous heparin as a prophylaxis for deep vein thrombosis and PE. Also, patients with a PE claim or any anticoagulant claim prior to the initial diagnosis date were excluded.

Eligible PE patients were stratified using the sPESI criteria into low-risk PE (LRPE) and high-risk PE (HRPE). The sPESI is a simplified version of the PESI in which selected variables of the original score are included (age, history of cancer, history of chronic cardiopulmonary disease, pulse, systemic blood pressure, and oxygen saturation levels). Patients scoring 0 points were considered at low risk. LRPE patients were further stratified, based on their LOS, into short LOS (≤2 days) and long LOS (>2 days) cohorts.

#### Methods and measurements

Patient demographics, including age, gender, race, and body mass index (BMI), during the baseline period were assessed. In addition, clinical characteristics, including Charlson comorbidity index (CCI) score, individual comorbidities (hospitalized DVT [ICD-9-CM codes 451.1, 453], left ventricular [LV] dysfunction [ICD-9-CM code 429.9], cardiac dysrhythmia [ICD-9-CM codes 427.0–427.9]) and administration of various diagnostic tests were recorded. Also, patients with various clinical markers including troponin I/T and natriuretic peptide testing results during the index hospitalization were assessed.

#### Outcomes

The percentage of patients with HACs and surgical procedures (thrombolysis, placement of inferior vena cava filter) during index hospitalization were evaluated. HACs were identified using the ICD-9-CM codes (S3 Table). APEs (recurrent venous thromboembolism [VTE], major bleeding, death), diagnostic tests (CTA, echocardiogram, lung ventilation/perfusion [VQ] scan, venous Doppler ultrasound) were also evaluated. Recurrent VTE was defined as having a hospitalization claim for DVT or PE between 8 and 90 days after the index date. Major bleeding was defined using a previously validated algorithm developed by Cunningham et al. [17]. The algorithm employed a systematic approach to identify major bleeding events using ICD-9-CM/CPT diagnosis and procedure codes (S2 Table). We defined the net clinical benefit as 1 minus the combined rate of APE events and HACs. The percentage of patients with any (ie, not disease-specific) inpatient hospitalization, outpatient stay, or pharmacy visit, as well as the mean number of visits per patient and the associated health care costs during the 90-day follow-up period, were reported.

#### Analysis

Descriptive statistics were provided for all study variables, including baseline demographics, clinical characteristics, and outcome variables among short LOS and long LOS LRPE cohorts, and statistical tests of significance (chi-square for categorical variables, t-test for continuous variables) were conducted to assess differences between the cohorts. Logistic regression was used to identify the predictors of hospital LOS (short vs long LOS) among LRPE patients. Patient characteristics such as gender, race, BMI, CCI score, baseline comorbidities, clinical markers, and diagnostic tests were included as independent variables in the model. Hospital LOS was the dependent variable. Odds ratio (OR) and 95% confidence intervals (CIs) were presented. Propensity score matching (PSM) was used to compare the clinical and economic outcomes among short vs long LOS LRPE cohorts. Each short LOS patient was matched to a long LOS patient within 0.01 units of the propensity score. The propensity score was calculated via a logistic regression model. The adequacy of the matching procedure was assessed by standardized difference; a difference of <10% is considered well balanced. Costs were compared between the PSM-matched cohorts with a generalized linear model with a gamma distribution and log link to account for the expected non-normality of cost data. All analyses were conducted using SAS statistical software (SAS 9.3, Copyright 2012, SAS Institute Inc., Cary, NC, USA.)

## Results

### Characteristics of study subjects

**Fig 1:**
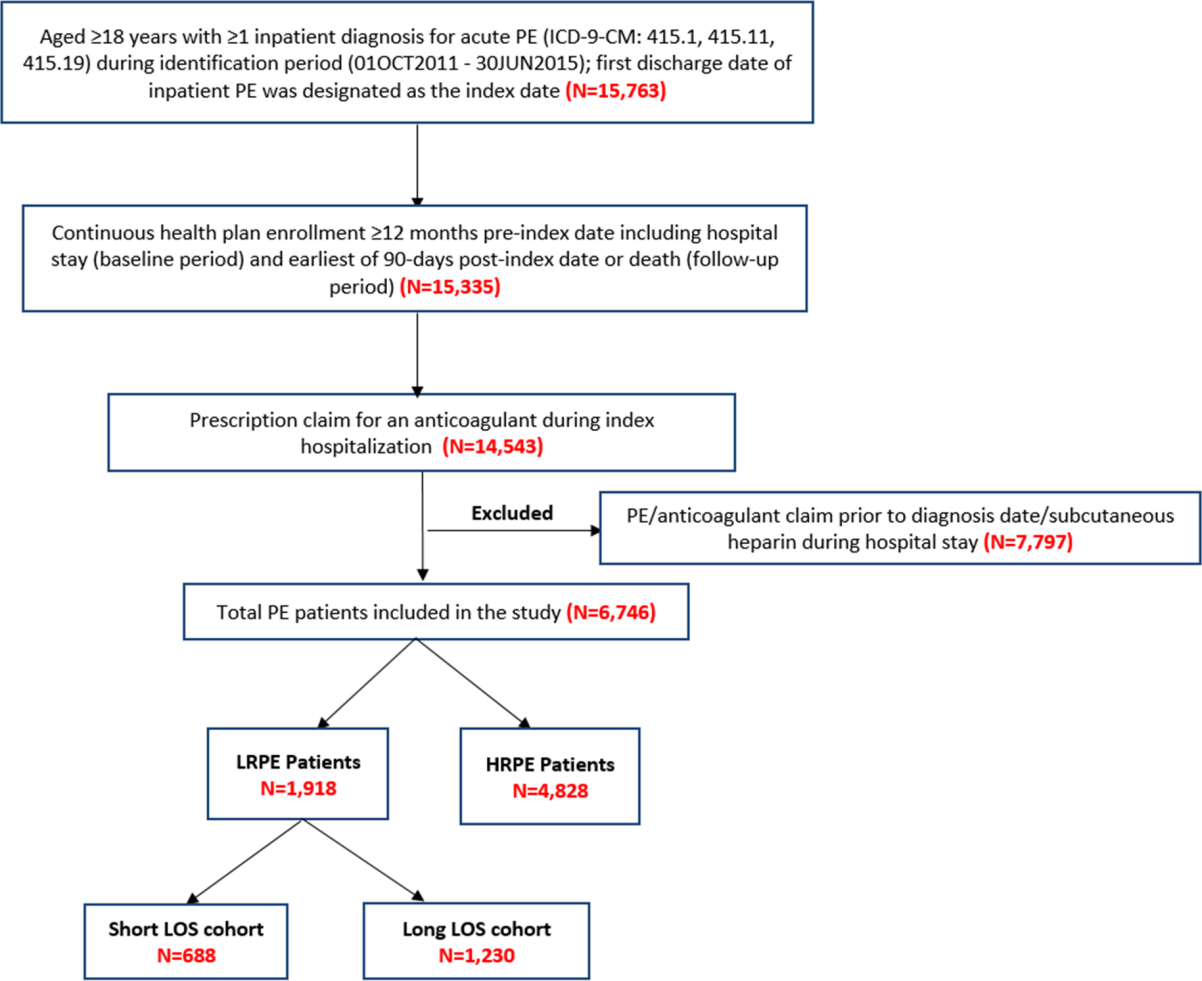
Study population and cohorts. HRPE: high risk pulmonary embolism; LOS: length of stay; LRPE: low-risk pulmonary embolism.

After applying the inclusion and exclusion criteria, 6,746 PE patients were included in the study. Among these patients, 1,918 (28.4%) met the definition of being LRPE patients. Among the LRPE patients, 688 (35.9%) had a short LOS and 1,230 (64.1%) had a long LOS (Fig 1).

Before matching, LRPE patients with a long LOS were older (60.7 vs 58.4 years, 95% CI: 1.28–3.37) and a higher percentage were men (94.6 vs 91.3, 95% CI: 1.01–1.06) as compared to LRPE patients with a short LOS. Long LOS patients had higher CCI scores (1.1 vs 0.8, 95% CI: 0.16–0.42) and a higher proportion of patients with individual baseline comorbidities, including moderate or severe renal disease (20.3% vs 14.2%, 95% CI: 1.04–1.96), diabetes (29.3% vs 22.5%, 95% CI: 1.10–1.53), and cardiac dysrhythmia (16.4% vs 10.0%, 95% CI: 1.27–2.12) as compared to short LOS patients. Also, the long LOS cohort had a higher proportion of patients with troponin I testing (38.1% vs. 30.1%, 95% CI: 1.11–1.45) and elevated troponin I >0.04 ng/ml (46.5% vs 23.7%, 95% CI: 1.11–1.45) or troponin T >0.03 ng/ml (73.7% vs 28.6%, 95% CI: 1.08–6.16) during index hospitalization as compared to the short LOS cohort (Table 1).

**Table 1:** Baseline demographic and clinical characteristics of LRPE patients with long versus short LOS.

	Long LOS		Short LOS (≤48 hrs) Cohort		95% Wald Confidence Limits	
	(>48 hrs) Cohort					
	N = (1,230)		N = (688)			
	N/	%/SD		N/			%/SD
	Mean			Mean			
**Age**						
Mean, SD	60.7	11.1	58.4	11.2	1.28	3.37
Median	63		61			
18–45	117	9.5%	100	14.5%	0.51	0.84
46–64	608	49.4%	346	50.3%	0.90	1.08
65+	505	41.1%	242	35.2%	1.03	1.32
**Sex**						
Male	1164	94.6%	628	91.3%	1.01	1.06
Female	66	5.4%	60	8.7%	0.44	0.86
**Race**						
White	786	63.9%	437	63.5%	0.94	1.08
Black	319	25.9%	187	27.2%	0.82	1.11
Unknown	94	7.6%	40	5.8%	0.92	1.88
Other	31	2.5%	24	3.5%	0.43	1.22
**Body Mass Index**						
Body Mass Index (kg/m^2^)	31.6	10.3	31.23	6.6	-0.49	1.21
**Baseline Comorbid Conditions**						
Charlson Comorbidity Index Score	1.1	1.5	0.8	1.3	0.16	0.42
Myocardial Infarction	67	5.5%	33	4.8%	0.76	1.70
Congestive heart failure	0	0.0%	0	0.0%		
Peripheral vascular disease	74	6.0%	40	5.8%	0.71	1.50
Dementia	15	1.2%	1	0.2%	1.11	63.38
Cerebrovascular disease	128	10.4%	45	6.5%	1.15	2.21
Chronic pulmonary disease	81	6.6%	49	7.1%	0.66	1.30
Rheumatologic disease or connective tissue disease	21	1.7%	7	1.0%	0.72	3.93
Peptic Ulcer disease	29	2.4%	4	0.6%	1.43	11.49
Mild liver disease	16	1.3%	8	1.2%	0.48	2.60
Hemiplegia or paraplegia	0	0.0%	0	0.0%		
Moderate or severe renal disease	250	20.3%	98	14.2%	1.04	1.96
Diabetes	360	29.3%	155	22.5%	1.10	1.53
Any tumor (Other Malignancy)	18	1.5%	4	0.6%	0.55	11.62
Moderate or severe liver disease	24	2.0%	6	0.9%	0.48	10.51
Metastatic solid tumor	0	0.0%	0	0.0%		
Diabetes + complications	178	14.5%	64	9.3%	1.05	2.30
AIDS	90	7.3%	42	6.1%	0.49	2.93
Cardiac Dysrhythmia	202	16.4%	69	10.0%	1.27	2.12
LV dysfunction	33	2.7%	6	0.9%	1.30	7.31
Hospitalized DVT	416	33.8%	208	30.2%	0.97	1.28
**Baseline Diagnostic Tests**						
CTA	453	36.8%	496	72.1%	0.47	0.56
ECHO	24	2.0%	13	1.9%	0.53	2.01
VQ Scan	23	1.9%	18	2.6%	0.39	1.32
Venous Doppler Ultrasound	210	17.1%	172	25.0%	0.57	0.82
**Clinical Marker during Index Hospitalization**						
# Patients with Troponin I, N	469	38.1%	207	30.1%	1.11	1.45
# Patients with Troponin T, N	19	1.5%	14	2.0%	0.38	1.50
# Patients with Natriuretic Peptide testing, N	453	36.8%	223	32.4%	1.00	1.29

CTA: Computed Tomography Angiography; DVT: deep vein thrombosis; ECHO: echocardiogram; LOS: length of stay; LRPE: low-risk pulmonary embolism; LV: left ventricular SD: standard deviation; VQ: lung ventilation/perfusion

After 1:1 PSM, a total of 784 (40.8%) patients were included in the long LOS (n = 392) and short LOS (n = 392) LRPE cohorts. The cohorts were well-balanced based on baseline demographic and clinical characteristics with standardized differences of < 10%.

### PSM-adjusted adverse PE events, HACs, surgical procedures, and diagnostic tests

There were no differences in the follow-up adverse PE events, including recurrent VTE, major bleeding, and death between the short LOS and long LOS LRPE cohorts. However, LRPE patients with a short LOS had fewer HACs (1.5% vs 13.3%, 95% CI: 3.77–19.94), including hospital-acquired pneumonia (0.0% vs 8.4%) during index hospitalization. Additionally, the short LOS cohort had a lower proportion of patients with bacterial pneumonia (5.9% vs 11.7%, 95% CI: 1.24–3.23) than the long LOS cohort. Net clinical benefit was higher for short LOS patients. (86.9% vs 78.3%, 95% CI: 0.84–0.96). Among long LOS, the number of HACs (52) exceeded adverse PE events (14 recurrent DVT, 5 bleeds, 10 deaths) (Table 2). LRPE patients with a long LOS had a higher number of surgeries for placement of the inferior vena cava filter than the short LOS patients (4.8% vs 0.8%, 95% CI: 1.89–21.23) during index hospitalization. During the 90-day follow-up period, the long LOS cohort, as compared to the short LOS cohort, had a lower proportion of patients with a VQ scan (1.8% vs 4.3%, 95% CI: 0.17–0.98) and a Venous Doppler Ultrasound (20.4% vs 29.6%, 95% CI: 0.54–0.88).

**Table 2:** PSM-adjusted hospital-acquired complications and adverse PE events among LRPE patients with long versus short LOS during the 90-day follow-up period.

	Long LOS		Short LOS (≤2 days) Cohort		95% Wald Confidence Limits*	
	(≥2 days) Cohort					
	N = (392)		N = (392)			
	N/Mean	%/SD	N/Mean	%/SD		
**Hospital-acquired Complications during Index Hospitalization**						
Hospital-acquired complications, any	52	13.3%	6	1.5%	3.77	19.94
Catheter-associated urinary tract Infection	2	0.5%	0	0.0%		
Methicillin-resistant staphylococcus aureus	5	1.3%	1	0.3%	0.59	42.60
*Clostridium difficile* infection	3	0.8%	0	0.0%		
Hospital-acquired (bacterial) pneumonia	33	8.4%	0	0.0%		
Foreign object retained after surgery	1	0.3%	0	0.0%		
Pressure ulcer stages III & IV	1	0.3%	0	0.0%		
Trauma/injury	13	3.3%	5	1.3%	0.94	7.22
Vascular catheter-associated infection	1	0.3%	0	0.0%		
Surgical site infection	1	0.3%	0	0.0%		
Bacterial pneumonia	46	11.7%	23	5.9%	1.24	3.23
**90-day Adverse PE Events**						
Recurrent VTE	14	3.6%	13	3.3%	0.51	2.26
Time to first VTE, days	34.6	26.7	34	18.3	-17.70	18.84
Major Bleeding	5	1.3%	4	1.0%	0.34	4.62
Time to first Major Bleeding, days	22.4	21.3	37	29.4	-54.42	25.22
Death	10	2.6%	10	2.6%	0.42	2.38
Time to Death, days	30.9	23.6	40.5	24	-31.99	12.79
**Net Clinical Benefit (1- [any hospital acquired complication or adverse PE events]**	307	78.3%	341	86.9%	0.84	0.96

* CI cannot be calculated if any one of the cohorts had 0.00% HAC's

PE: pulmonary embolism; PSM: propensity score matching; SD: Standard Deviation; VTE: venous thromboembolism

#### PSM-adjusted follow-up health care resource utilization and costs

**Fig 2:**
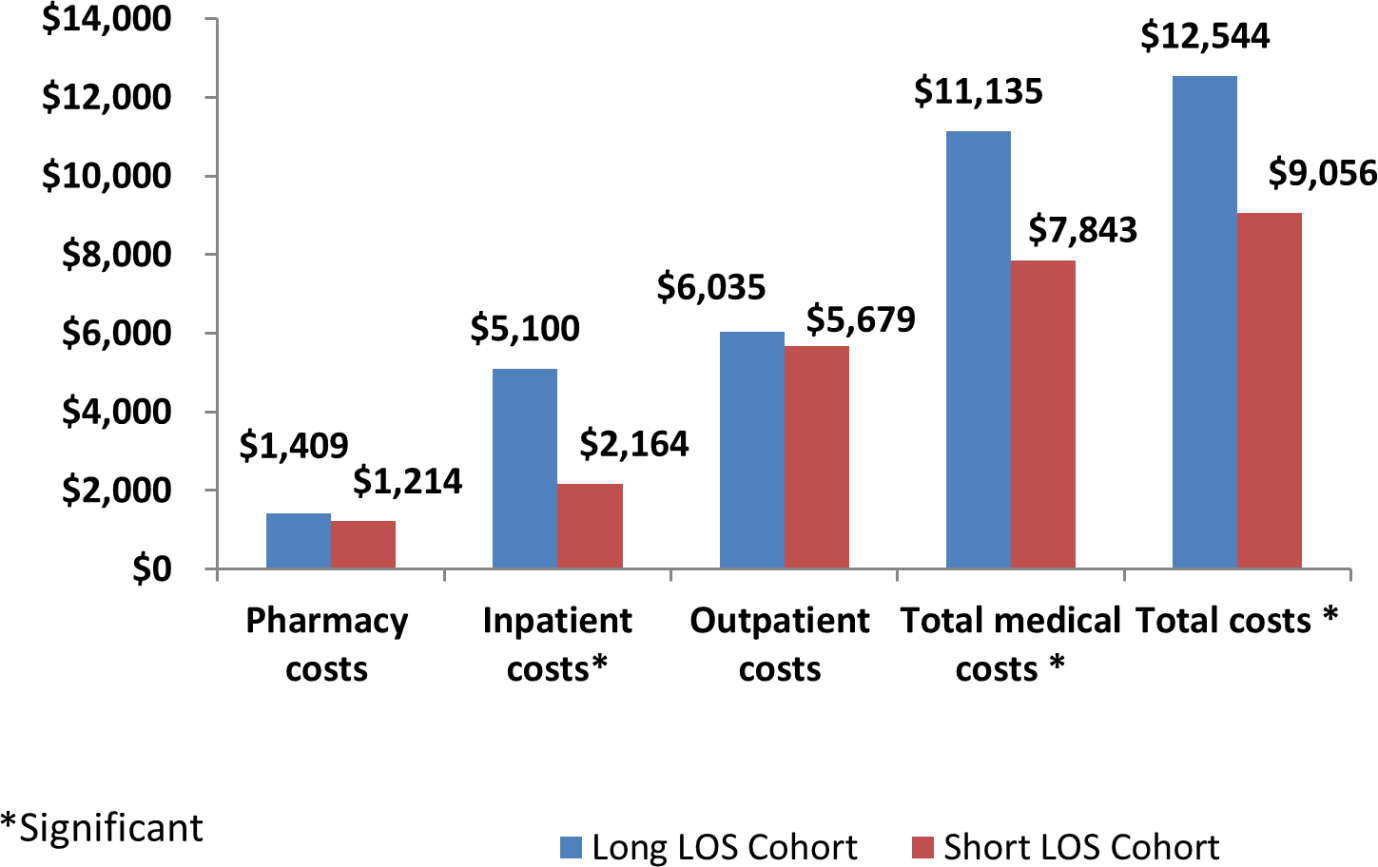
PSM-adjusted health care costs among LRPE patients with long vs short LOS in the 90-day follow-up period. LOS: length of stay.

The long LOS cohort, as compared to the short LOS cohort, had a higher number of pharmacy visits per patient (12.2 vs 9.4, 95% CI: 1.34–4.18) and incurred higher inpatient ($5,100 vs $2,164, 95% CI: $646.80-$5225.00), total medical ($11,135 vs $7,843, 95% CI: $796.40-$5787.70) and total costs ($12,544 vs $9,056, 95% CI: $636.60-$6337.70; Fig 2).

#### Predictors of hospital LOS among LRPE patients

Patients with CTA (OR: 4.8, 95% CI: 3.8–6.0), VQ scan (OR: 3.8, 95% CI: 1.9–7.7), and venous Doppler ultrasound (OR: 1.4, 95% CI: 1.1–1.9) in the baseline period had increased probability of a short LOS among LRPE patients, and patients with assays for clinical markers troponin I (OR: 0.7, 95% CI: 0.5–0.9) and natriuretic peptide testing (OR: 0.7, 95% CI: 0.6–0.9) during index hospitalization had decreased probability of a short LOS. The odds of a short LOS decreased among LRPE patients with LV dysfunction (OR: 0.2, 95% CI: 0.1–0.6), hospitalized DVT (OR: 0.7, 95% CI: 0.6–0.9), and peptic ulcer disease (OR: 0.3, 95% CI: 0.1–1.0).

## Limitations

The findings from our study should be viewed in the context of some study limitations. First, the study relied on retrospective claims data. While claims data are extremely valuable for the efficient and effective examination of health care outcomes, treatment patterns, and costs, they are collected for payment and not research. The presence of a diagnosis code on medical claims is not a positive presence of disease and may be incorrectly coded or included as rule-out criteria rather than the actual disease. To ensure exclusion of any rule-out PE diagnosis, PE patients were required to have an anticoagulant claim during their hospital stay. The presence of a claim for a filled prescription does not indicate the medication was consumed or taken as prescribed. Also, prescriptions filled over-the-counter or provided as samples by the physician are not observed in claims data. Thus, the true number of medications prescribed may not be accurately recorded. Third, certain clinical and disease-specific parameters are not readily available in claims data that could have effect on study outcomes.

Patients in the long LOS cohort may have had non-coded reasons explaining their longer stay. For example, those with unstable social situations may have been kept longer while attempting to arrange more stable outpatient management strategies. Further, longer LOS patients may have been perceived to suffer greater fragility by their physicians, which may explain their older age. Finally, it should be noted that troponin results are not included in the majority of PE risk stratification scores. Thus, higher troponin levels found in the longer LOS cohort may have prompted additional evaluation that required a longer inpatient time or may have prompted a longer admission as some studies suggest higher risk with elevated biomarkers. Interestingly, our study does not confirm worse outcomes in patients with elevated biomarkers.

Finally, it should also be noted that PSM adjustment cannot resolve problems due to imbalances in unmeasured factors. It is possible that there were unobserved variables that the PSM does not correct for in risk-adjusted tables. The current study also represented only US data from a specific subpopulation (VHA veterans), who were mostly elderly men. Therefore, the general applicability of our findings to young male patients or females requires further study.

## Discussion

PE is one of the leading causes of cardiovascular morbidity and mortality [18]. The key to effective treatment lies in the appropriate risk stratification–low-risk patients can benefit from abbreviated hospital stays or outpatient therapy, which can reduce the economic burden of the disease [19]. In our study, we found that in a real-world clinical setting, LRPE patients with a short LOS have better clinical outcomes at lower costs than those with a long LOS. Importantly, LRPE patients with longer LOS suffered markedly higher rates of hospital acquired conditions. Our comparison between long and short LOS patients is unique and allows the quantification of the impact of hospital exposure in putatively comparable populations.

In a study conducted by Dentali et al., 26.1% of PE patients were classified as low-risk, while 30.7% were classified as low-risk by Jimenez et al., per the sPESI criteria [20,21]. Our results were consistent with these studies, since 28.4% of PE patients were stratified as low-risk. Results of our study showed that CTA was the most common diagnostic test used among the 2 cohorts, which is consistent with previous studies [22,23]. Surprisingly, the short LOS cohort included a higher proportion of patients with a CTA test than the long LOS cohort. This may have been due to the possibility that health care providers were confronted with diagnostic uncertainty and thus performed more tests to confirm the diagnosis.

In our analysis, patients with a longer LOS also had higher mean troponin levels. Some PE risk stratification tools include troponin, levels but the data is inconclusive on the risk associated with elevations therefore we included patients with elevations in the LRPE cohort. This is in contradistinction to a study conducted by Kang et al., in high-risk patients who had elevated levels of cardiac biomarkers including troponin T/I [24]. Additionally, in a meta-analysis published by Becattini and colleagues on the relation between troponins and mortality and morbidity in acute PE, they confirmed that the increase of troponins I and/or T was associated with higher medical complications and mortality even in the subgroup of low-risk and hemodynamically stable patients but not higher PE related adverse events [25]. This evidence supports the findings of our study as we observed elevated clinical markers in the LRPE patients with a longer LOS, but suggests this marker may be less relevant than clinical risks. Given the nature of the study methodology, we cannot discern whether or not patients with elevated troponin had a longer LOS because clinicians were executing additional diagnostics in this subpopulation. Despite differences in study design/settings and study population, our finding of increased HACs among long LOS patients is consistent with a number of previous studies [26,27,28]. However, it is difficult to determine whether a longer LOS is caused by medical complications or that a longer LOS caused the complications, as pointed out in earlier studies [26,27]. This challenge is also present in our study and should be kept in mind when interpreting the findings. The results of a meta-analysis conducted by Zondang et al. showed that PE patients who were treated at home or those discharged early had similar pooled incidences of recurrent VTE and major bleeding as those patients who were treated in the hospital. The study also observed that PE-related mortality did not occur in either the patients treated at home or discharged early [29]. The results of our study agree with the above study, as we observed no differences in the follow-up clinical outcomes including recurrent VTE, major bleeding, and death between the short LOS and long LOS LRPE patients.

Our study showed that long LOS patients had more inpatient and pharmacy visits and higher health care costs. Previous studies showed that increased hospital LOS is an important driver of costs among PE patients and suggested that implementation of outpatient treatment strategies or early discharge would substantially reduce health care costs [22,30, 31]. Studies have suggested that the home treatment of PE has cost savings in the range of $500–$2,500 per patient [7,32]. Additionally, Coleman et al. observed lower costs of hospital treatment among low-risk PE patients discharged within 2 or 3 days than those who stayed longer in the hospital [8].

Recently-published practice guidelines that recommend outpatient care for carefully selected patients with non-massive PE fail to specify how these low-risk patients can be identified accurately [32]. Previous studies observed that PE patients with a shorter LOS had higher post-discharge mortality, implying that the physicians may inappropriately select those patients at a higher risk for complications for early discharge [30,33]. Hence, outpatient and early discharge should only be considered in low-risk PE patients [29]. sPESI criteria used for risk stratification in our study is the most extensively-validated clinical prognostic criteria [9,21]. The results of a cohort study conducted by Donadini et al. showed that LOS was only associated with national early warning score (NEWS), a non-specific PE validated score, where NEWS ≥5 was considered a negative predictor for shorter LOS [34]. Additionally, previous studies assessing the patient and hospital factors in hospitalized patients showed that the presence of a DVT, higher rates of individual comorbidities, and a higher risk score were more likely to have a longer LOS among PE patients [30,32]. Our study results are in agreement with these studies, since patients with hospitalized DVT, peptic ulcer disease, and clinical markers troponin I and B natriuretic peptide were negative predictors for a shorter LOS. However, more research is necessary to evaluate the association between LOS and diagnostic tests. There is a need to conduct further studies assessing HACs and the associated health care utilization and costs among LRPE patients. Further research will help to provide efficient treatment and thus improve outcomes and reduce health care costs among PE patients.

In summary, the results of this study showed that LRPE patients with a short LOS had better clinical outcomes at lower costs than those with a long LOS. Therefore, risk stratification of PE patients is of utmost importance, and reducing the LOS among LRPE patients may substantially reduce the disease’s clinical and economic burden.

## Supporting information

S1 Fig Computer algorithm for identifying bleeding-related hospitalizations. (DOCX)

S1 Table ICD-9-CM diagnoses indicating bleeding of the type, according to site. (DOCX)

S2 Table Diagnosis codes indicating bleeding when confirmed by a secondary discharge diagnosis in S1 Fig or a code indicating transfusion. (DOCX)

S3 Table ICD-9-CM for hospital acquired complications. (DOCX)
