# Supplementary material for: Benefit of early discharge among patients with low-risk pulmonary embolism
Source: PLoS One. 2017 Oct 10;12(10):e0185022. doi: 10.1371/journal.pone.0185022 (PMC5634547; doi:10.1371/journal.pone.0185022)
Supplement: S1 Fig — (DOCX) [file pone.0185022.s001.docx]

S1 Figure. Computer Algorithm for Identifying Bleeding-related Hospitalizations[^[[1]](#endnote-1)^]

Hospitalizations with two types of primary diagnoses were considered: those indicating a bleed with no further confirmation needed (S1 Table), and those indicating a bleed (S2 Table) when confirmed by either a hospital revenue code indicating transfusion/cross-matching for transfusion or presence of a secondary discharge diagnosis in S1 Table.

The algorithm did not consider hospitalizations in which bleeding is considered as a secondary diagnosis, as these hospitalizations were more likely to identify bleeding that began in the hospital or due to other causes.

1. Cunningham A, Stein CM, Chung CP, Daugherty JR, Smalley WE, Ray WA. An automated

   database case definition for serious bleeding related to oral anticoagulant use.

   Pharmacoepidemiol Drug Saf. 2011;20(6): 560-566. [↑](#endnote-ref-1)
