# Supplementary material for: Benefit of early discharge among patients with low-risk pulmonary embolism
Source: PLoS One. 2017 Oct 10;12(10):e0185022. doi: 10.1371/journal.pone.0185022 (PMC5634547; doi:10.1371/journal.pone.0185022)
Supplement: S1 Table — (DOCX) [file pone.0185022.s002.docx]

S1 Table. ICD-9-CM Diagnoses Indicating Bleeding of the Type, According to Site

| Code | Diagnosis | Code | Diagnosis |
| --- | --- | --- | --- |
| *Gastroduodenal Site* | | *Lower Gastrointestinal Site* | |
|  |  | 455.2 | Internal hemorrhoids with other complication |
| 531.0x | Acute gastric ulcer with hemorrhage | 455.5 | External hemorrhoids with other complication |
| 531.2x | Acute gastric ulcer with hemorrhage and perforation | 455.8 | Unspecified hemorrhoids with other complication |
| 531.4x | Chronic or unspecified gastric ulcer with hemorrhage | 562.02 | Diverticulosis of small intestine with hemorrhage |
| 531.6x | Chronic or unspecified gastric ulcer with hemorrhage and perforation | 562.03 | Diverticulitis of small intestine with hemorrhage |
| 532.0x | Acute duodenal ulcer with hemorrhage | 562.12 | Diverticulosis of colon with hemorrhage |
| 532.2x | Acute duodenal ulcer with hemorrhage and perforation | 562.13 | Diverticulitis of colon with hemorrhage |
| 532.4x | Chronic or unspecified duodenal ulcer with hemorrhage | 568.81 | Hemoperitoneum |
| 532.6x | Chronic or unspecified duodenal ulcer with hemorrhage and perforation | 569.3 | Hemorrhage of rectum and anus |
| 533.0x | Acute peptic ulcer, site unspecified, with hemorrhage | 569.85 | Angiodysplasia of intestine with hemorrhage |
| 533.2x | Acute peptic ulcer, site unspecified, with hemorrhage and perforation | *Unspecified Gastrointestinal Site* | |
| 533.4x | Chronic peptic ulcer, site unspecified, with hemorrhage | 578.1 | Blood in stool |
| 533.6x | Chronic peptic ulcer, site unspecified, with hemorrhage and perforation | 578.9 | Hemorrhage of gastrointestinal tract, unspecified |
| 534.0x | Acute gastrojejunal ulcer with hemorrhage |  |  |
| 534.2x | Acute gastrojejunal ulcer w hemorrhage/perforation |  |  |
| 534.4x | Chronic gastrojejunal ulcer with hemorrhage | *Genitourinary Site* | |
| 534.6x | Chronic gastrojejunal ulcer with hemorrhage and perforation | 593.81 | Vascular disorders of kidney |
| 535.01 | Acute gastritis with hemorrhage | 599.7 | Hematuria |
| 535.11 | Atrophic gastritis with hemorrhage | 623.8 | Other specified non-inflammatory disorders of vagina |
| 535.21 | Gastric mucosal hypertrophy with hemorrhage | 626.2 | Excessive/frequent menstruation, with secondary diagnosis indicating acute bleeding: anemia (280.0,285.1,285.9), orthostasis (458.0), syncope (780.2) |
| 535.31 | Alcoholic gastritis with hemorrhage |  |  |
| 535.41 | Other specified gastritis with hemorrhage | 626.6 | Metrorrhagia |
| 535.51 | Unspecified gastritis and gastroduodenitis with hemorrhage | *Cerebral Site* | |
| 535.61 | Duodenitis with hemorrhage | 430 | Subarachnoid hemorrhage |
| 537.83 | Angiodysplasia of stomach and duodenum with hemorrhage | 431 | Intracerebral hemorrhage |
|  |  | 432.0 | Nontraumatic extradural hemorrhage |
| *Esophageal Site* | | 432.1 | Subdural hemorrhage |
| 456.0 | Esophageal varices with bleeding | 432.9 | Unspecified intracranial hemorrhage |
| 456.20 | Esophageal varices in diseases classified elsewhere with bleed |  |  |
| 530.7 | Mallory-Weiss tear (gastroesophageal laceration-hemorrhage syndrome) | *Other Site* | |
| 530.82 | Esophageal hemorrhage | 423.0 | Hemopericardium |
|  |  | 459.0 | Hemorrhage, unspecified |
|  |  | 568.81 | Hemoperitoneum (nontraumatic) |
| *Upper Gastrointestinal, Unspecified* | | 719.1x | Hemarthrosis |
| 578.0 | Hematemesis | 784.7 | Epistaxis |
|  |  | 784.8 | Hemorrhage from throat |
|  |  | 786.3 | Hemoptysis |
