# Supplementary material for: Benefit of early discharge among patients with low-risk pulmonary embolism
Source: PLoS One. 2017 Oct 10;12(10):e0185022. doi: 10.1371/journal.pone.0185022 (PMC5634547; doi:10.1371/journal.pone.0185022)
Supplement: S2 Table — (DOCX) [file pone.0185022.s003.docx]

S2 Table. Diagnosis Codes Indicating Bleeding When Confirmed by a Secondary Discharge Diagnosis in S1 Figure or a Code Indicating Transfusion. *

| Code | Diagnosis | Code | Diagnosis |
| --- | --- | --- | --- |
| *Gastroduodenal Site* | | *Lower Gastrointestinal Site* | |
| 531.1 | Gastric ulcer acute with perforation | 455.x | Hemorrhoids, not incl. 455.2,455.5, 455.8 |
| 531.3 | Acute gastric ulcer without mention of hemorrhage or perforation | 562.00 | Diverticula of small intestine without mention of hemorrhage |
| 531.5 | Chronic or unspecified ulcer with perforation | 562.01 | Diverticulitis of small intestine without mention of hemorrhage |
| 531.7 | Chronic gastric ulcer without mention of hemorrhage or perforation | 562.10 | Diverticula of colon without mention of hemorrhage |
| 531.9 | Gastric ulcer, unspecified, without mention of hemorrhage or perforation | 562.11 | Diverticulitis of colon without mention of hemorrhage |
| 532.1 | Duodenal ulcer acute with perforation |  |  |
| 532.3 | Acute duodenal ulcer without mention of hemorrhage or perforation | *Esophageal Site* | |
| 532.5 | Duodenal ulcer chronic/unspecified w perforation | 530.1 | Esophagitis |
| 532.7 | Duodenal ulcer chronic without mention of hemorrhage or perforation |  |  |
| 532.9 | Unspecified as acute or chronic duodenal ulcer without mention of hemorrhage or perforation | *Unspecified Site* | |
| 533.1 | Peptic ulcer acute with perforation | 280.0 | Anemia due to loss of blood** |
| 533.3 | Peptic ulcer, acute, no mention of hemorrhage | 285.1 | Acute posthemorrhagic anemia |
| 533.5 | Peptic ulcer chronic/unspecified w perforation | 285.9 | Anemia, unspecified** |
| 533.7 | Peptic ulcer, chronic, without mention of hemorrhage or perforation | 790.92 | Abnormal coagulation profile** |
| 533.9 | Peptic ulcer, unspecified as acute or chronic without mention of hemorrhage or perforation |  |  |
| 534.1 | Gastrojejunal ulcer acute with perforation |  |  |
| 534.3 | Gastrojejunal ulcer acute without mention of hemorrhage or perforation |  |  |
| 534.5 | Gastrojejunal ulcer chronic/unspecified w perforation |  |  |
| 534.7 | Chronic gastrojejunal ulcer without mention of hemorrhage or perforation |  |  |
| 534.9 | Gastrojejunal ulcer, unspecified acute: chronic, without mention of hemorrhage or perforation |  |  |
| 535.00 | Acute gastritis without mention of hemorrhage |  |  |
| 535.10 | Atrophic gastritis, no mention of hemorrhage |  |  |
| 535.20 | Gastric mucosal hypertrophy without mention of hemorrhage |  |  |
| 535.30 | Alcoholic gastritis, no mention of hemorrhage |  |  |
| 535.40 | Other specified gastritis, no mention hemorrhage |  |  |
| 535.50 | Unspecified gastritis and gastroduodenitis without mention of hemorrhage |  |  |
| 535.60 | Duodenitis without mention of hemorrhage |  |  |

Note: The sites for primary and secondary diagnoses do not need to match.

*Hospital revenue codes indicating transfusion or processing of blood products for transfusion

**These codes are only accepted if there is a secondary diagnosis for confirmation; a transfusion is not sufficient.

Investigation of profiles for these codes with transfusions suggested they primarily were persons with chronic

anemia and the admissions were not for an acute bleeding episode.
