# Supplementary material for: Benefit of early discharge among patients with low-risk pulmonary embolism
Source: PLoS One. 2017 Oct 10;12(10):e0185022. doi: 10.1371/journal.pone.0185022 (PMC5634547; doi:10.1371/journal.pone.0185022)
Supplement: S3 Table — (DOCX) [file pone.0185022.s004.docx]

**S3 Table. ICD-9-CM for Hospital Acquired Complications**

| **Hospital Acquired Complications** | **IC-9-CM Codes** |
| --- | --- |
| Catheter-associated urinary tract infection | 996.64 |
| Methicillin-resistant staphylococcus aureus | 038.12, 424.42, 041.12 |
| Clostridium difficile infection | 008.45 |
| Hospital acquired (bacterial) pneumonia | 481, 482, 483, 485, 486, 507 |
| Foreign object retained surgery | 998.4,998.7 |
| Air embolism | 999.1 |
| Blood incompatibility | 999.60, 999.61, 999.62, 999.63, 999.69 |
| Pressure ulcer stage III, IV | 707.23,707.24 |
| Trauma/injury |  |
| Fracture | 800-829 |
| Dislocation | 830-839 |
| Intracranial injury | 850-854 |
| Crushing injury | 925-929 |
| Burn | 940-949 |
| Electric shock | 991-994 |
| Procedure-related | E876.5, E876.6, E876.6 |
| Poor glycemic control | 250.10-250.13, 250.20-250.23, 251.0, 249.10-249.11, 249.20-249.21 |
| Iatrogenic pneumothorax with venous catheterization | 512.1, 38.93 |
| Vascular catheter-associated infection | 999.31, 999.32, 999.33 |
| Surgical site infection |  |
| Mediastinitis, following coronary artery bypass graft | 519.2 and one of the following procedure codes, 36.10-36.19 |
| Surgical site infection following certain orthopedic procedures | 996.67, 998.59 and one of the following procedure codes 81.01-81.08, 81.23, 81.24, 81.31-81.38, 81.83 or 81.85 |
| Surgical site infection following bariatric surgery for obesity | 278.01, 539.01, 539.81, 998.59 and one of the following procedure codes: 44.38, 44.39, 44.95 |
| Surgical site infection following cardiac implantable electronic device | 996.61, 998.59 and one of the following procedure codes: 00.50-00.54, 37.80-37.83, 37.85-37.87, 37.94, 37.96, 37.98, 37.74, 37.75, 37.76, 37.77, 37.79 or 37.89 |
| Bacterial Pneumonia | 481, 482, 483, 485, 486 and 507 |

Note: Any condition with no patients was not reported in the results table.
